# Urothelial Carcinoma Recurrence With an Ileal Conduit: Multimodal Management With Extirpative Surgery, Chemotherapy, and Immunotherapy

**DOI:** 10.7759/cureus.51157

**Published:** 2023-12-27

**Authors:** Parth U Thakker, Justin Refugia, Maxwell Sandberg, Alejandro R Rodriguez, Ashok K Hemal

**Affiliations:** 1 Urology, Atrium Health Wake Forest Baptist Medical Center, Winston-Salem, USA

**Keywords:** radical cystectomy, avelumab, metastatic urothelial carcinoma, ileal conduit resection, urothelial carcinoma recurrence

## Abstract

Ileal conduit (IC) is the most performed urinary diversion after radical cystectomy (RC) for urothelial carcinoma (UC) of the bladder. While UC recurrence after RC is well-described, recurrence of UC within a urinary diversion is much less prevalent, and thus, management of these lesions is not well understood. Here, we report the case of a 59-year-old male with a history of invasive UC with glandular differentiation of the urinary bladder who had carcinoma in situ recurrence after induction, intravesical Bacille Calmette-Guerin therapy. He underwent robot-assisted laparoscopic radical cystoprostatectomy (RALC) with bilateral pelvic lymph node dissection and intracorporal ileal conduit (IC) urinary diversion. Two years later, he presented to the emergency department with hematuria. Computed tomography demonstrated a mass within the IC. He subsequently underwent IC resection and ligation of bilateral ureters and had permanent nephrostomy tubes placed, with the final pathology confirming high-grade UC. Positron emission tomography revealed hypermetabolic soft tissue implants within the greater omentum and retroperitoneum for which he underwent fine-needle aspiration, demonstrating recurrence of poorly differentiated UC. Ultimately, the patient started treatment with systemic gemcitabine and carboplatin and completed 4 cycles before transitioning to maintenance avelumab therapy. No disease progression was noted at 16 months post-treatment. Herein, we present a review of the literature and our management of the present patient.

## Introduction

Ileal conduit (IC) is the most performed urinary diversion after radical cystectomy (RC) for urothelial carcinoma (UC) of the bladder [1]. The risk of pelvic recurrence after RC has been reported to range from 5-15% within 2 years with distant recurrences in up to 50% of patients [2]. Conversely, upper tract and urethral recurrences after RC have been reported to be up to 6.4% and 6%, respectively [1,3]. Management of these recurrences has been well described.

The recurrence of UC within a urinary diversion is much less prevalent and thus, the management of these lesions is not well understood. These recurrences are primarily detected with the presence of hematuria and positive cytology with or without loopography and upper tract imaging, and the management of these recurrences has ranged from endoscopic management to radical resection. Regardless of the treatment modality, survival after this recurrence remains poor. However, improved management of advanced UC may have an impact on the management of these patients [2].

Herein, we present a patient with a recurrence of poorly differentiated UC within an IC two years after RC for refractory carcinoma in situ (CIS), who subsequently received palliative gemcitabine and carboplatin with maintenance avelumab without progression of disease at 16 months.

## Case presentation

A 52-year-old male was initially diagnosed with CIS and non-muscle invasive bladder cancer (NMIBC) with glandular differentiation. He underwent maximal transurethral resection of the bladder tumor and intravesical Bacille Calmette-Guerin (BCG) therapy and remained disease-free for 6 years. At the age of 58, a surveillance computerized tomography (CT) scan demonstrated a polypoid lesion in the bladder. Transurethral resection at that time demonstrated diffuse CIS recurrence. The patient elected to undergo radical cystoprostatectomy (RALC) with pelvic lymph node dissection (PLND) and intracorporeal IC, which was uneventful with negative distal ureteral margins by way of intraoperative frozen sections, and his hospital course was unremarkable. The final pathology was pTisN0 with negative surgical margins and an incidental Grade Group 1 prostatic adenocarcinoma (pT2N0) was found. Nineteen months after surgery, the patient developed a right-sided ureteroenteric stricture requiring placement of an externalized nephroureteral stent, which was subsequently exchanged for an indwelling, single-J stent.

Two years post-RALC, he presented with gross hematuria and anemia and CT demonstrated bilateral hydronephrosis and an 11.2 cm mass within the IC. He did not have any ascites. Permanent bilateral nephrostomy tubes were placed. Conduit cytology was obtained and was consistent with high-grade carcinoma. Staging imaging was negative for metastatic disease. After a discussion of management options, the patient elected to proceed with the resection of the conduit.

**Figure 1:**
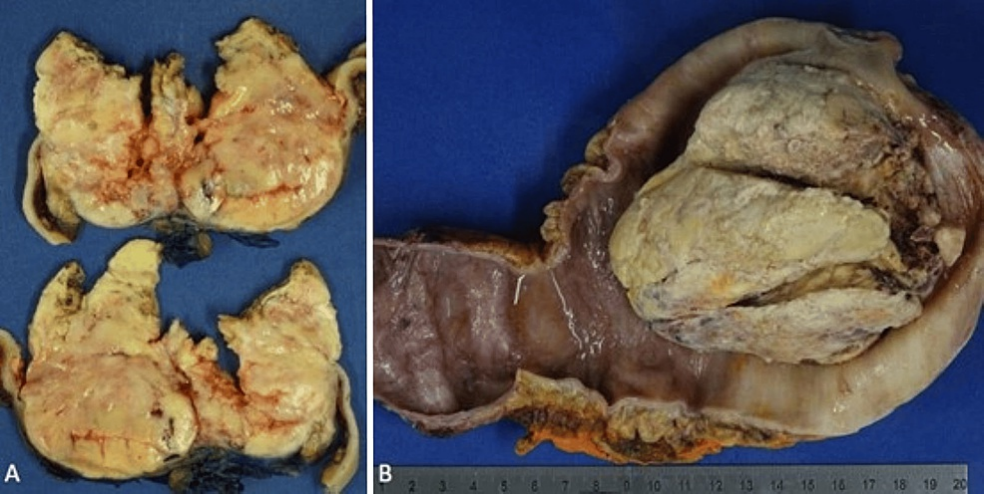
Gross imaging (A) Representative cross-sections of the mass isolated from the ileal conduit with soft, friable, hemorrhagic components. (B) Mass within the proximal end of the ileal conduit with marked dilation of the conduit.

**Figure 2:**
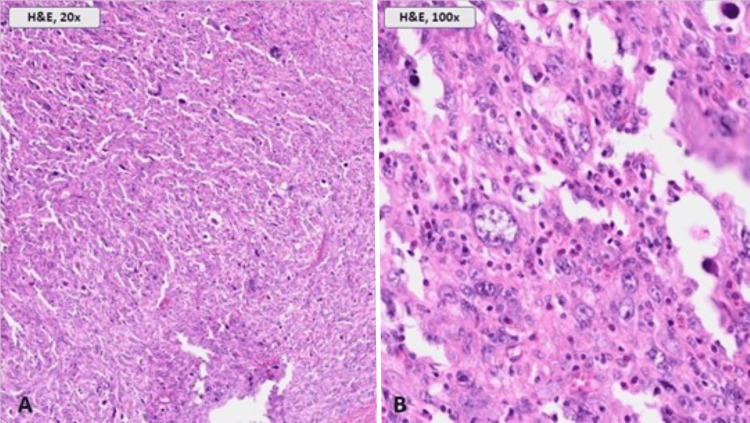
Microscopic analysis (A) Sheets of highly pleomorphic, malignant cells in a background of inflammatory and degenerated cells (H&E, 20x). (B) High magnification view of the malignant cells, highlighting marked cytologic atypia with significant nuclear pleomorphism, prominent nucleoli, and mitotic activity (H&E, 100x).

**Figure 3:**
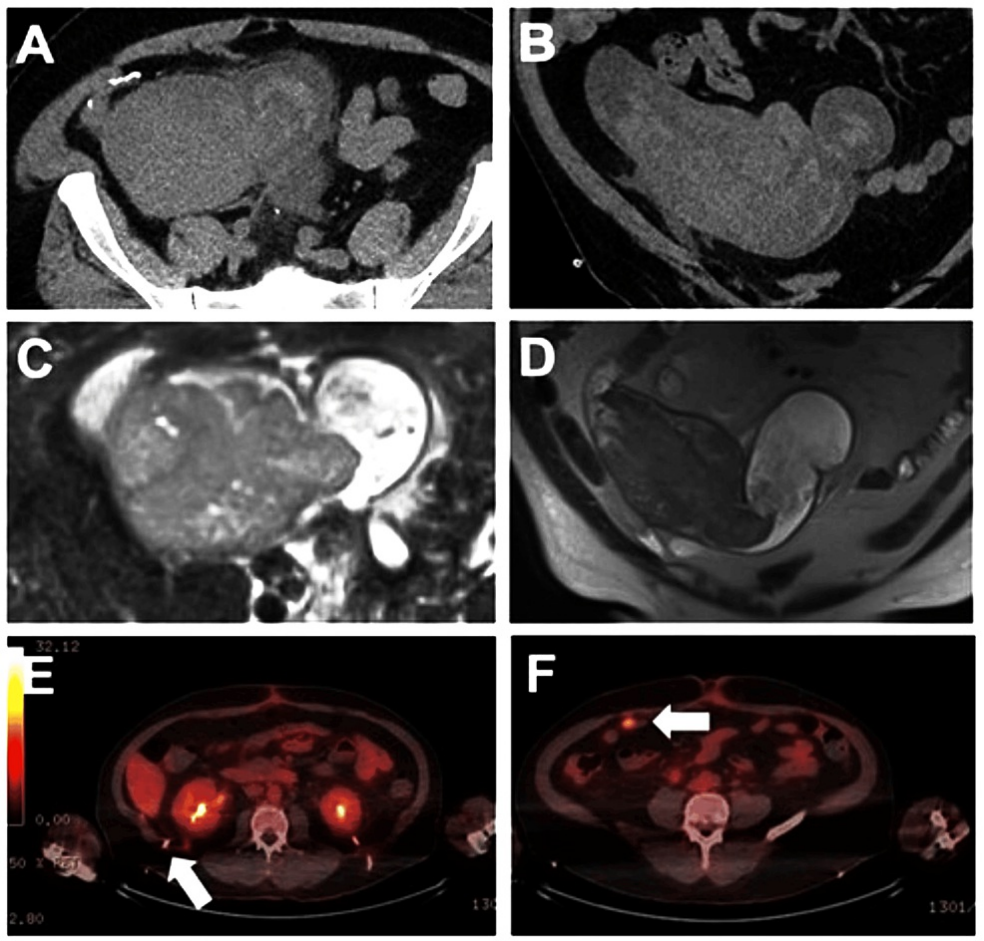
Cross-sectional imaging Representative cross-sectional imaging with CT (A&B), and MRI (C&D) with a mass in the ileal conduit. Arrows in panels E and F demonstrate, 19F FDG PET images with right hypermetabolic retroperitoneal (E), and omental soft-tissue implant (F). Interventional radiology-guided biopsy was performed on the retroperitoneal implant depicted in E.

The ileal conduit was resected via a midline laparotomy incision. Dense adhesions to the small bowel and omentum made the resection precarious. Attempts were made to dissect the ureters, free of the diversion, and create another diversion. However, the ureters were ultimately ligated bilaterally, due to extreme friability of tissue. The final pathology of the IC mass was poorly differentiated UC with negative surgical margins (Figure 1, 2). Separately submitted samples of the omentum and bilateral ureters were negative for malignancy. The diagnosis of the mass was further confirmed by immunohistochemistry showing that the tumor cells were positive for cytokeratin AE1/AE3, weakly positive for GATA3, and negative for S100, PAX8, NKX3.1, CDX2, TTF-1, and p63. These findings are consistent with recurrent UC within an IC. Two months post-operatively, a surveillance positron emission tomography (PET) scan demonstrated hypermetabolic soft tissue implants within the greater omentum and retroperitoneal lymphadenopathy consistent with disease progression (Figure 3E, 3F). The omentum was biopsied which showed pure, poorly differentiated UC. He then underwent fine needle aspiration (FNA) of the retroperitoneal lesion (Figure 3A), the pathology of which was pure, poorly differentiated UC. The patient was not a candidate for palliative cisplatin therapy due to renal impairment and was thus initiated on a regimen of systemic gemcitabine (800mg/m2) and carboplatin (AUC 4). Treatment was discontinued after 4 cycles due to recurrent episodes of pyelonephritis, cellulitis, anemia, and thrombocytopenia. However, as interval CT imaging demonstrated a complete response, he was then transitioned to maintenance avelumab which he has been tolerating well. Currently, 16 months after resection, the patient is alive and without evidence of disease progression.

## Discussion

Recurrence of UC in an IC without involvement of the upper urinary tract is uncommon, particularly when presenting with metastatic disease. These recurrences carry a poor prognosis, with recurrence-free and overall survival of 15.8% and 24.2%, respectively, at 18 months in one study [3]. While exceedingly rare, very few reported cases have implemented platinum-based chemotherapy and no cases to date have utilized immunotherapy in the management of these patients. To our knowledge, few cases of recurrence within an IC have been reported in the literature and a majority of the cases report patient death before 2 years of follow-up [4]. While the cause of UC recurrence in urinary diversion after RC is unclear, several theories have been previously proposed. The first theory is a local invasion into the diversion from an undiagnosed tumor at the ureteral margin. The second theory is the implantation of exfoliated upper tract tumor cells in the intestinal segment at the time of diversion. However, a third possibility is by hematogenous or lymphatic spread [5].

The etiology of recurrence in this patient remains unclear as this patient had negative ureteral margins at the time of RALC. Additionally, hematogenous and lymphatic drainage patterns are distinct between the small bowel and bladder. Thus, recurrence may be most likely because of undiagnosed or microscopic upper urinary tract disease insofar as the presence of undiagnosed CIS of the upper tracts. Involvement of the greater omentum on the PET-CT appears to indicate local invasion through the urinary diversion rather than a metastatic site. However, the retroperitoneal adenopathy in this case was indicative of non-regional metastasis from the initial disease site.

The initial management of UC recurrences within urinary diversions has included endoscopic, partial, and complete resection of the urinary diversion [6]. Implementation of chemotherapy in the palliative setting has not been common, however, most reported cases have utilized cisplatin-based regimens. To our knowledge, the use of carboplatin-based chemotherapy regimens in these patients has not been reported given the well-understood superiority of cisplatin-based therapy [6-8]. While quality studies comparing the efficacy of carboplatin versus cisplatin are lacking, combination therapy with gemcitabine and carboplatin has been shown to be non-inferior to the M-CAVI (methotrexate, carboplatin, and vinblastine) regimen for those patients unfit for cisplatin-based therapy [9]. Additionally, maintenance avelumab has been demonstrated to improve overall survival compared to supportive care alone in patients without progression of metastatic UC on initial therapy [10]. Given the appropriate response this patient has exhibited thus far, we believe the 5-year survival for these patients may rival that of patients with more commonly seen metastatic UC of the urinary bladder. 

## Conclusions

In the present case, we described an instance of an aggressive UC recurrence within an IC, two years after RC for refractory CIS. This finding is a rare occurrence, and the prognosis tends to be poor. The management pattern we describe for this patient, however, has been successful thus far. We elected to have him undergo resection of the urinary diversion, where he subsequently developed metastatic UC. He was treated with first-line gemcitabine and carboplatin followed by maintenance avelumab. Only conventional CT scans have been performed to date after starting avelumab. Nevertheless, this current regimen has provided progression-free survival at 16 months.
